# Impact of COVID-19 Lockdown on Screen Time and Sleep Quality Among Medical and Allied-Health Undergraduates in Chennai: A Cross-Sectional Study

**DOI:** 10.7759/cureus.94899

**Published:** 2025-10-19

**Authors:** Akila Govindarajan Venguidesvarane, Samya Varadarajan, Muthukumar Rajamohan, Saranya Varadarajan

**Affiliations:** 1 Community Medicine, Sri Ramachandra Institute of Higher Education and Research, Chennai, IND; 2 Oral Pathology, Saveetha Dental College, Chennai, IND

**Keywords:** covid-19 lockdown, public health, screen time, sleep quality, students

## Abstract

Background

The COVID-19 pandemic and subsequent lockdowns disrupted the daily routine of students. Increased screen exposure and altered sleep patterns emerged as potential long-term health concerns during this period. This study aimed to assess screen time and sleep quality among undergraduate students before and during the COVID-19 lockdown.

Methods

A cross-sectional study was conducted among 470 final-year undergraduate students in a medical teaching college and research institute in India from July to December 2020. Data were collected using a pre-tested semi-structured questionnaire, which included screen time, sleep quality assessed using the Pittsburgh Sleep Quality Index (PSQI), and gaming addiction using the Internet Gaming Disorder Scale-Short Form (IGDS9-SF). Information was obtained for periods before and during the lockdown. Statistical analyses included the Wilcoxon signed-rank test, the McNemar-Bowker test, and the multivariable logistic regression.

Results

The median screen time increased significantly during the lockdown compared to before (8 hours vs. 5 hours; p < 0.001). Usage of social media, streaming platforms (OTT (over-the-top)) platforms, and gaming also increased. The prevalence of sleep problems rose from 14.3% before lockdown to 23.2% during lockdown (p < 0.05). Logistic regression revealed a dose-response association between screen time and poor sleep quality: compared with <4 hours, adjusted odds ratios (AOR) were 3.3 (95%CI: 1.2-9.0) for 4-8 hours, 2.8 (95%CI: 0.98-8.1) for 8-12 hours, and 3.9 (95%CI: 1.3-11.6) for >12 hours. Depressive symptoms and higher fear of COVID-19 scores were also significant predictors of poor sleep.

Conclusions

The COVID-19 lockdown significantly increased screen time and sleep disturbances among undergraduate students. Monitoring digital media usage and providing appropriate advice on the importance of sleep are critical during public health crises to promote overall health and functioning among undergraduates.

## Introduction

Sleep plays an integral role in the maintenance of growth, development, homeostasis, and physical and mental health of individuals [1-3]. Though sleep problems among the elderly population are emphasized more [4], the recent trends show that disturbances in sleep are becoming more frequent among youngsters, especially young adults. Currently, as reported by the National Sleep Foundation, the ideal sleep requirement of an adolescent aged 14-17 years is 8-10 hours a day, for young adults between 18 and 25 years is around 7-9 hours a night to maintain a healthy lifestyle, basic optimal health, and development [5]. Addressing sleep-related behaviors in young adults is essential, given the growing evidence of its impact on health and daily functioning.

Inadequate sleep or sleep disturbance has become a common problem in young adults globally [6,7]. As previously discussed, sleep insufficiency in quantity and quality has a direct relationship with physical and mental wellness that leads to depression, behavioural issues, excessive daytime drowsiness, and metabolic dysfunctions. The previously reported causes for sleep disturbances in young adults have been attributed to family, social, and environmental factors; however, recent trends show the impact of digital media and excess screen time on the quality of sleep [6].

The increased screen time in young adults could be attributed to the increased availability in recent years. This leads to addiction, which in turn causes behavioural changes and affects sleep. In India, 92.8% of the members own a mobile phone, and 35% of the population has access to the internet, as reported in 2017 [8,9]. The age group that uses the internet the most is 12-29 years, which is more than two-thirds (67%) of internet users [10].

The World Health Organization (WHO) estimates that more than 38 million people die each year from non-communicable diseases [11]. Encouraging healthy lifestyle practices among medical and paramedical undergraduates is instrumental in creating a healthy cohort of future healthcare providers [12].

Social networks have gained a lot of popularity among students, especially in the pandemic period, as it is their only source of entertainment, education, and connection with peers [13]. More than 350 social networking websites are operational across the web [14]. In spite of the abundant availability, the data regarding the purpose of use is scarce, especially in adult education in the medical and paramedical fields. According to a case study done in Bangladesh, it was observed that around 96% of students who had access to the internet made use of social networking platforms like chatting, texting, blogging, Myspace, Facebook, etc., and 71% of them were using these on a weekly basis [15]. The present generation is highly comfortable using digital media, showing a declining interest in learning through libraries and books [16].

Internet and digital media usage have witnessed a significant surge during the COVID-19 pandemic. The lockdown limited students’ social interactions, physical activity, and education, while disrupting their sleep and increasing the time spent on electronic devices [17-19]. Worldwide, 35 million people were affected by COVID-19, and one million succumbed to it. Isolation and closure of educational institutions significantly impacted students’ mental health [20,21]. Experience from prior public health crises and pandemics reveals the association of negative emotions among people and knowledge gaps on disease transmission mechanisms can deeply impact the preventive strategies aimed by the public health department to curb the disease spread [22].

Educational institutions were compelled to find ways to help students manage their emotional wellbeing and continue with their academics in the absence of in-person teaching. Coping with the COVID-19 pandemic was influenced by augmenting physical activity levels, which play a key role in augmenting immunity, significantly lowering anxiety and stress, and improving sleep, thereby playing a crucial role in the overall wellbeing of individuals [23].

Therefore, this study aimed to compare screen time and sleep quality among undergraduate students in the medical and allied health sciences before and during the COVID-19 lockdown, and to identify predictors of poor sleep, including psychological factors such as depressive symptoms, stress, and fear of COVID-19.

## Materials and methods

This study was conducted among the undergraduate students from the Bachelor of Medicine, Bachelor of Surgery (MBBS), Bachelor of Dental Surgery (BDS), Bachelor of Physiotherapy (BPT), and Bachelor of Pharmacy (B. Pharm) programs at Sri Ramachandra Institute of Higher Education and Research (SRIHER), Chennai, Tamil Nadu, India. The study was conducted after obtaining approval from the Sri Ramachandra Institute of Higher Education and Research (approval number: IEC-NI/20/OCT/76/94). Written informed consent was obtained from all participants before the study commencement.

Study participants

All final year undergraduate students who consented to the study were included. All students in the final year of the MBBS, BDS, BPT, and B. Pharm programs who were willing to participate in the study were included in the study. Students who did not provide consent to participate, those who were undergoing their internship, and those enrolled in other academic years were excluded from the study.

Sample size and sampling method

We conducted a complete enumeration (census) of all final-year MBBS, BDS, BPT, and B. Pharm students who were eligible and consented; therefore, no a priori sample size calculation was performed. The number of eligible participants was 550 (250 MBBS, 100 BDS, 100 BPT, and 100 B Pharm students). All of them were individually approached. A total of 470 students consented to participate. For transparency, with n=470, the two-sided 95% confidence interval (CI) around an observed proportion near 23% (poor sleep during lockdown) has an approximate half-width of ±3.6 percentage points, indicating adequate precision for prevalence estimates in this cohort.

Recruitment and data collection

After obtaining permission from the lead authority of each college, an informed consent was sent to all the students in the final year. Those students who consented were scheduled for a one-on-one interview to reduce reporting bias and improve response rate. A pilot study was conducted before the commencement of the data collection to ensure reliability. Data was collected through in-person interviews with the students between July and December 2020. Participants were asked to recall their typical screen use and sleep patterns during the month immediately preceding the national lockdown in March 2020 (“pre-lockdown period”) for comparison with behaviors during the lockdown period. This study was done during the lockdown period with retrospective recall of pre-lockdown behaviours. All participants responded to every question; hence, there was no missing data.

Demographic Details

The first part of the questionnaire (see Appendices) included demographic data like age, gender, etc. Physical activity level was self-reported and categorized as low, moderate, or high based on frequency and duration of exercise or sports activity per week. Body mass index (BMI) was measured in person using a standard portable stadiometer and digital weighing scale during the lockdown period, following COVID-19 safety protocols.

Assessment of Screen Time

A questionnaire was designed that was structured in nature, and pre-testing was done to determine the total screen time of the study participants. Screen time was operationally defined as the total daily duration (in hours) spent using electronic devices (mobile phones, tablets, laptops, and television) for entertainment, social interaction, and study. For each category (social media, OTT (over-the-top) platforms, gaming), participants reported average daily duration before and during lockdown. Data on changes in time spent on screen in gaming, OTT, and social media platforms before the lockdown period and during the lockdown period were obtained. The Internet Gaming Disorder Scale-Short Form (IGDS9-SF) was used to determine if the study participants suffered from gaming addiction or disorder. The IGDS9-SF, an unidimensional tool, has nine items that reflect the respective nine criteria for IGD as in the DSM-5. Scoring was done based on the responses of the participants, and the scores were added to get the total score. The total scores range between 9 and 49. Greater scores indicate a greater degree of disorder [24].

Assessment of Sleep

Sleep quality was measured using the Pittsburgh Sleep Quality Index (PSQI), which assesses seven components: subjective sleep quality, sleep latency, sleep duration, habitual sleep efficiency, sleep disturbances, use of sleep medication, and daytime dysfunction, yielding a global score from 0 to 21 (higher = worse sleep). Consistent with standard practice, we defined poor sleep as PSQI >5 and good sleep as ≤5 [25].

Assessment of Depression

Depressive symptoms were assessed using the Patient Health Questionnaire-9 (PHQ-9) [26]. This nine-item instrument asks respondents to rate the frequency of depressive symptoms over the past two weeks on a four-point Likert scale (0 = not at all to 3 = nearly every day). The total score ranges from 0-27 and is categorized as minimal (0-4), mild (5-9), moderate (10-14), moderately severe (15-19), and severe (20-27). The PHQ-9 has been widely validated in student and Indian populations, with good internal consistency (Cronbach’s α >0.80) [27]

Assessment of Fear of COVID-19

Fear related to COVID-19 was measured using the Fear of COVID-19 Scale (FCV-19S) [28], a seven-item validated instrument. Each item was rated on a five-point Likert scale (1 = strongly disagree to 5 = strongly agree), with total scores ranging from 7 to 35. Higher scores indicate greater fear of COVID-19. For analysis, we used the total score as a continuous variable. The FCV-19S has been validated in multiple countries, including India, and demonstrates good internal reliability (Cronbach’s α ≈0.85) [29].

Assessment of Stress

Perceived stress was measured using the four-item Perceived Stress Scale (PSS-4), a validated short version of the original PSS. Each item was rated on a five-point Likert scale ranging from 0 (“never”) to 4 (“very often”). The total score was obtained by summing all four items, yielding a range from 0 to 16, with higher scores reflecting greater perceived stress. In this study, PSS-4 was analysed as a continuous variable (mean ± SD) [30].

Data analysis

Statistical analysis was done using IBM SPSS Statistics for Windows, version 16 (IBM Corp., Armonk, New York, United States). Background variables, assessment of screen time, and sleep were presented using descriptive statistics as numbers and percentages. For continuous/ordinal variables that were not normally distributed (e.g., screen time, social media use, OTT use, gaming), the Wilcoxon signed-rank test was used to compare medians between pre- and during-lockdown periods. For categorical variables related to sleep quality and its components, paired comparisons were made using McNemar’s test (binary) and McNemar-Bowker test (multi-category). Associations between independent variables (sociodemographic and psychosocial factors) and poor sleep were initially assessed using Chi-square tests. A multivariable binary logistic regression model was built to identify independent predictors of poor sleep quality during lockdown. Adjusted odds ratios (AORs) with 95% CIs were calculated. Prior to logistic regression, multicollinearity was assessed using linear regression collinearity diagnostics. Tolerance values >0.7 and VIF <2.0 indicated no significant multicollinearity. Model performance and discriminatory ability were evaluated using the receiver operating characteristic (ROC) curve analysis. The area under the curve (AUC) with 95% CI was reported. A p-value <0.05 was considered statistically significant.

## Results

A total of 470 students were included in the study, of whom 62.3% were females. Table 1 shows that students had a significant increase in median screen time during lockdown compared with recalled screen usage before lockdown (median 8 hours, interquartile range (IQR) 6-10 hours versus median 5 hours, IQR 4-8 hours; p < 0.001). Similar significant increases were observed in various domains of screen usage: social media (median 4 hours, IQR 2-5 hours versus median 2 hours, IQR 1-3 hours), OTT platforms (median 3 hours, IQR 1.7-5 hours versus median 1 hour, IQR 1-2 hours), and gaming (median 1 hour, IQR 0-3 hours versus median 0 hours, IQR 0-1 hour).

**Table 1:** Median duration of screen usage in the study population MD: median difference; OTT: over the top

Variables	Lockdown Period, median (IQR)	Pre-Lockdown Period, median (IQR)	Hodges–Lehmann Estimate of the MD	Wilcoxon Signed-Rank Test, Z score	Ranked biserial r	p value
Total screen time	8 (6,10)	5(4,8)	2	-16.570	0.764	<0.0001
Social media usage	4 (2,5)	2(1,3)	1	-16.276	0.750	<0.0001
OTT usage	3 (1.7,5)	1(1,2)	1	-14.836	0.683	<0.0001
Gaming	1 (0.0,3)	0(0.0,1)	0	-11.835	0.545	<0.0001

Table 2 shows the distribution of the study population based on the duration of screen time. About 15.5% of the study population reported screen usage for more than 12 hours during lockdown, when compared to their recalled pre-lockdown screen usage of 2.8%. There was a shift in the duration of screen time to the higher side across all domains, like social media, OTT, and gaming. Specifically, the number of students using social media for over eight hours increased from 1% before lockdown to 5.1% during lockdown. The duration of usage of OTT and gaming was less when compared to social media usage, both before and during lockdown.

**Table 2:** Duration of screen usage before and during lockdown (N=470) OTT: over the top

Categories (hours/day)	Total screen time (Lockdown), n (%)	Total screen time (Before lockdown), n (%)	Social media use (Lockdown), n (%)	Social media use (Before lockdown), n (%)	OTT use (Lockdown), n (%)	OTT use (Before lockdown), n (%)	Gaming (Lockdown), n (%)	Gaming (Before lockdown), n (%)
<4	57 (12.1)	161 (34.3)	300 (63.8)	406 (86.4)	349 (74.3)	437 (93.0)	422 (89.8)	459 (97.7)
4-8	221 (47.0)	228 (48.5)	146 (31.1)	59 (12.6)	108 (23.0)	31 (6.6)	42 (8.9)	9 (1.9)
8-12	119 (25.3)	68 (14.5)	18 (3.8)	2 (0.4)	13 (2.8)	2 (0.4)	5 (1.1)	1 (0.2)
>12	73 (15.5)	13 (2.8)	6 (1.3)	3 (0.6)	0 (0)	0 (0)	1 (0.2)	1 (0.2)

Table 3 shows that overall, 83.8% of study participants reported that their screen time increased during lockdown. Similarly, 75.1%, 64.7% and 36.8% of students reported that their social media usage, OTT usage, and gaming increased during lockdown. There was no change in the duration of screen time usage, social media usage, OTT, and gaming in 12.8%, 22.6%, 32.8%, and 61.7% of study participants, respectively. A smaller percentage (1.5-3.4%) reported decreased usage across screen domains.

**Table 3:** Change in screen usage before and during lockdown (N=470) OTT: over the top

Variables	Increased, n (%)	Decreased, n (%)	No change, n (%)
Total screen time	394 (83.8)	16 (3.4)	60 (12.8)
Social media usage	353 (75.1)	11 (2.3)	106 (22.6)
OTT change	304 (64.7)	12 (2.6)	154 (32.8)
Gaming	173 (36.8)	7 (1.5)	290 (61.7)

Gaming addiction was found in 1.1% of the study participants according to IGDS9-SF. Among those with gaming addiction, it was found that 2.8% of the male participants had addiction, and none of the female participants were addicted; 50% of the study population reported that online gaming reduces stress. Using games as a stress reduction strategy was seen almost equally among male (50.6%) and female (49.4%) participants.

Sleep problems and related variables

Table 4 represents sleep problems before and during lockdown. Overall, the prevalence of sleep problems increased during lockdown to 23.2% when compared to before lockdown, 14.3%. A significant decline in sleep quality was observed, with 45.3% of the study population reporting “very good” sleep quality during lockdown, when compared to 62.8% of students before lockdown. About 29.2% of the study population took two to three hours to fall asleep (sleep latency) during lockdown, when compared to 10% before lockdown (p < 0.001).

**Table 4:** Details of sleep problems before and during lockdown (N=470) PSQI: Pittsburgh Sleep Quality Index

Sleep problems	Lockdown Period, n (%)	Pre-lockdown Period, n (%)	McNemar–Bowker test χ^2^, P value
Poor sleep (PSQI >5)	109 (23.2)	67 (14.3)	19.1, <0.001
Sleep quality			79.17, <0.0001
Very good	213 (45.3)	295 (62.8)	
Fairly good	194 (41.3)	158 (33.6)	
Fairly bad	46 (9.8)	12 (2.6)	
Very bad	17 (3.6)	5 (1.1)	
Sleep latency (minutes)			133.6, <0.0001
≤15	168 (35.7)	282 (60)	
16–30	165 (35.1)	141 (30	
31–60	91 (19.4)	41 (8.7)	
>60	46 (9.8)	6 (1.3)	
Sleep duration (hours)			145.39, <0.0001
>7	356 (75.7)	185 (39.4)	
6-7	92 (19.6)	249 (53.0)	
5-6	13 (2.8)	30 (6.4)	
<5	9 (1.9)	6 (1.3)	
Habitual sleep efficiency			24.02, 0.01
>85%	413 (87.9)	377 (80.2)	
75-84%	27 (5.7)	61 (13.0)	
65-74%	20 (4.3)	18 (3.8)	
<65%	10 (2.1)	14 (3.0)	
Sleep disturbance			38.5, <0.0001
None	136 (28.9)	184 (39.1)	
Mild	313 (66.6)	270 (57.4)	
Moderate/ severe	21 (4.5)	16 (3.4)	
Sleep medication			10.43, 0.064
None	445 (94.7)	454 (96.6)	
Occasional	15 (3.2)	9 (1.9)	
Frequent	7 (1.5)	6 (1.3)	
Very frequent	3 (0.6)	1 (0.2)	
Daytime dysfunction			61.11, <0.0001
None	258 (54.9)	308 (65.5)	
Mild	135 (28.7)	126 (26.8)	
Moderate	65 (13.8)	32 (6.8)	
Severe	12 (2.6)	4 (0.9)	

Interestingly, the duration of sleep and habitual sleep efficiency were better during lockdown when compared to before lockdown. However, about 39.1 % of the study population slept without any disturbance before lockdown, when compared to 28.9% of students during lockdown (p < 0.001). Similarly, sleep medication and daytime dysfunction were more common during lockdown when compared to before lockdown.

Factors Associated with Poor Sleep

Table 5 shows the association of various background variables with sleep problems during lockdown. In bivariate analysis, male gender, higher BMI, and low physical activity were not significantly associated with poor sleep quality. However, screen time showed a clear dose-response relationship; compared with students reporting <4 hours, those with 4-8 hours (OR 3.5, 95%CI: 1.3-9.2), 8-12 hours (OR 3.1, 95%CI: 1.1-8.4), and >12 hours (OR 4.2, 95%CI: 1.4-11.9) had significantly higher odds of poor sleep.

**Table 5:** Factors associated with poor sleep during lockdown Ref: reference category; – : not applicable; OR: odds ratio; AOR: adjusted odds ratio; CI: confidence interval; PHQ9: Patient Health Questionnaire-9

Variables	Poor sleep, n (%)	Good sleep, n (%)	OR (95%CI)	p value	AOR (95%CI)	p value
Gender						
Female	67 (22.9)	226 (77.1)	1(Ref)	-	1(Ref)	-
Male	42 (23.7)	135 (76.3)	1.1 (0.6-1.6)	0.830	0.9 (0.5-1.5)	0.777
BMI						
<18.5	4 (17.4)	19 (82.6)	1 (Ref)	-	1(Ref)	-
18.5-22.9	37 (20.0)	148 (80.0)	1.1 (0.3-3.7)	0.767	0.8 (0.2-2.4)	0.683
23-27.5	50 (25.6)	145 (74.4)	1.6 (0.5-5.0)	0.390	1.1 (0.4-3.6)	0.775
>27.5	17 (27.4)	45 (72.6)	1.7 (0.5-6.0)	0.345	1.5 (0.4-4.9)	0.544
Physical activity						
Moderate	78 (22.7)	265 (77.3)	1 (Ref)	-	1 (Ref)	-
Low	31 (24.4)	96 (75.6)	1.1 (0.6-1.7)	0.703	0.97 (0.6-1.6)	0.897
Screen time						
<4	5 (8.8)	52 (91.2)	1 (Ref)	-	1 (Ref)	-
4-8	56 (25.3)	165 (74.7)	3.5 (1.3-9.2)	0.011	3.3 (1.2-9)	0.019
8-12	27 (22.7)	92 (77.3)	3.1 (1.1-8.4)	0.031	2.8 (0.98-8.1)	0.056
>12	21 (28.8)	52 (71.2)	4.2 (1.4-11.9)	0.007	3.9(1.3-11.6)	0.015
PHQ9						
Minimal	14 (9.9)	128 (90.1)	1(Ref)	-	1(Ref)	-
Mild	30 (23.6)	97 (76.4)	2.8 (1.4-5.6)	<0.001	2.7 (1.3-5.5)	0.080
Moderate	37 (28.9)	91 (71.1)	3.7 (1.9-7.2)	<0.001	3.8 (1.8-7.9)	<0.001
Moderate severe	21 (41.2)	30 (58.8)	6.4 (2.9-14.02)	0.044	5.4 (2.3-12.8)	<0.001
Severe	7 (31.8)	15 (68.2)	4.2 (1.4-12.2)	0.021	3.7 (1.2-12.1)	0.030
Fear of corona scale, mean±SD	13.41 ± 5.06	12.33 ± 4.8	1.2 (1.1-1.3)	0.044	1.06 (1.01-1.11)	0.030
Perceived stress scale, mean±SD	7.46 ± 2.4	6.76 ± 2.8	1.2 (1.1-1.3)	0.021	1.04 (0.94-1.14)	0.464

A logistic regression model was used for the predictors of poor sleep. All predictors had variance inflation factor (VIF) values < 2 (range: 1.02-1.27) and tolerance > 0.7, indicating the absence of multicollinearity. The maximum condition index was 21.1, well below the threshold of 30. Thus, no multicollinearity issues were detected. On multivariable logistic regression, screen time of four to eight hours (AOR 3.3, 95%CI: 1.2-9.0) and >12 hours (AOR 3.9, 95%CI: 1.3-11.6) remained significant predictors. Increasing severity of depressive symptoms was strongly associated with poor sleep, with the odds highest among those with moderate to severe depression (AOR 5.4, 95%CI: 2.3-12.8). Fear of COVID-19 also emerged as a significant factor (AOR 1.06, 95%CI: 1.01-1.11), while perceived stress was not independently associated after adjustment.

ROC analysis showed that the logistic regression model had an AUC of 0.572 (95%CI: 0.513-0.631, p = 0.023), indicating poor but statistically significant discriminatory ability in predicting poor sleep during lockdown.

## Discussion

This study evaluated the changes in screen time and sleep quality among undergraduate students during the COVID-19 lockdown. The study observed a significant increase in screen time during the lockdown period. We also observed that the sleep problems among the students were significantly higher during the lockdown. Similar to our findings, the global trends also highlight the rise in sleep disturbances among young adults in recent years [1-3]. Sleep is essential for physical, psychological, and emotional health, and its disruption can have widespread consequences [4]. Though the National Sleep Foundation recommends seven to nine hours of sleep for young adults [5], changes in modern lifestyle and excessive digital media usage lead to insufficient sleep and irregular sleep patterns [6,7]. Our study supports this trend, linking excess screen time during lockdown with increased sleep problems.

In today’s world, digital devices have become an integral part of the daily routines of young adults, especially among the 12-29-year-olds [10], and this accounts for over 67% of the media users, making university students vulnerable to the adverse effects of screen exposure. In the context of the pandemic, media usage and sleep disturbances have intensified significantly, altering students’ daily routine [17-19]. Such disruptions affect the physical, mental, and emotional health of students [20,21].

The results of our study align with many others. About 15.5% of our study population had an excess screen time of over 12 hours a day during lockdown, compared to a recalled screen time of 2.8% before lockdown. There was a sharp rise in social media usage as well. This is similar to the findings by Hedderson et al., who reported a similar rise in screen usage during the pandemic among children [31]. Though the study population is different, the findings imply that increased screen usage affects young populations due to the need for remote learning environments. The findings are concurrent with those of Ganesh et al., who reported increased screen time usage in medical and engineering students during lockdown [32].

Gaming addiction was low in our study (1.1%), but more than half of the participants reported that gaming could reduce their stress levels. This shows that youngsters perceive gaming as a coping mechanism to reduce stress, especially during situations like the lockdown period. With multiple stressors like fear of infections, disturbed daily routines, and lack of in-person interactions, the shift to digital gaming can occur. Students may not be fully aware of the long-term consequences of excessive screen time and its correlation with sleep and mental wellbeing.

Studies have shown that increased screen exposure disturbs circadian rhythm and melatonin production, resulting in delayed sleep onset and poor sleep quality [33,34]. In our study, sleep problems were reported in 23.2% of students during lockdown compared to 14.3% before lockdown. These findings are consistent with Dongol et al., who reported that over 30% of college students experienced clinical insomnia during the pandemic [35]. Our study also reported that poor sleep was significantly associated with excess screen time >4 hours. Apart from the digital behaviours, psychological factors also significantly affect sleep. Higher levels of depression and increased fear of COVID-19 are some of the psychological factors associated with poor sleep. The associations emphasise the intricate interplay of behavioural and emotional factors during public health crises [36]. During lockdown, students reported some improvements in sleep duration and habitual sleep efficiency, likely because reduced academic and social schedules allowed them to spend more time in bed. However, this was accompanied by poorer subjective sleep quality, longer sleep latency, and more sleep disturbances. These changes suggest that while students slept longer, they felt their sleep was less refreshing and had more difficulty initiating sleep. Increased stress, irregular routines, and greater use of digital devices at night may have contributed to this. Similar patterns of longer sleep but poorer quality during COVID-19 lockdowns have been observed in a study by Cellini et al. [37].

To further assess the predictive ability of our logistic regression model, we constructed an ROC curve. The ROC analysis yielded an AUC of 0.572, indicating limited discriminatory capacity. In practical terms, this means that the model correctly distinguishes between individuals with and without sleep disturbances only slightly better than chance. Although statistically significant, the model’s discriminatory power was moderate, suggesting that factors included in the model explain sleep disturbances only partially. Reporting this finding is still valuable, as it highlights the complexity of sleep problems and the need for more comprehensive models. Future studies should incorporate additional psychosocial and behavioural variables to improve predictive accuracy

As sleep plays a crucial role in cognitive functions, emotional regulation, and homeostasis, identification and reduction of factors affecting sleep, especially during pandemics, is essential. Educational interventions and wellness programs are needed to facilitate healthy digital habits and sleep for undergraduate students. 

Strengths and limitations

The study involved a relatively large sample size and included all medical and allied health, and paramedical students. The tools used to assess screen time were pre-tested, and we used a validated tool to assess sleep. Validated instruments were also used to assess depressive symptoms, stress, and fear of COVID-19, enhancing reliability. The analysis applied appropriate non-parametric and multivariable methods, accounting for potential collinearity.

However, there were some limitations. As students were recruited from multiple disciplines within the university, the results can be generalized to undergraduate students within the medical and allied health streams. However, generalizability to other academic domains like engineering and the arts remains limited. A major limitation is recall bias, since pre-lockdown screen time and sleep parameters were self-reported retrospectively. This design does not allow causal inference, and findings should be interpreted with caution. Although it allowed rapid assessment, the study design does not fully capture temporal changes as a longitudinal study would. Future studies should consider prospective designs or sensitivity analyses based on the timing of recall. We were unable to perform sensitivity analyses by time since lockdown due to a lack of date stratification. Additionally, physical activity was self-reported, which may introduce measurement bias. The study could not account for potential residual confounding factors such as caffeine intake, academic workload, or type of device used, which may have influenced sleep outcomes.

The modest explanatory power of the regression model (AUC = 0.57) further suggests that unmeasured psychosocial and environmental factors may contribute to sleep disturbances beyond those captured in our analysis. Moreover, because the lockdown period was associated with multiple simultaneous lifestyle changes, including altered routines, stress, physical activity, and social interaction, it is not possible to completely isolate the independent effect of increased screen time on sleep quality. The varying recall interval between early and late respondents (4-9 months) may also have introduced additional recall variability.

## Conclusions

COVID-19 lockdown was associated with a marked increase in screen time and changes in sleep patterns among undergraduate students. While sleep duration and efficiency appeared to improve, students reported poorer subjective sleep quality, longer sleep latency, and more disturbances. Higher screen use, particularly social media, OTT platforms, and gaming, showed a dose-response relationship with poor sleep. Psychological factors, including depressive symptoms and fear of COVID-19, further contributed to sleep problems. These findings highlight the need for preventive strategies that promote balanced digital use, mental well-being, and healthy sleep routines among students, particularly future healthcare professionals, during public health crises. Going forward, integrating digital health literacy and sleep hygiene education into post-pandemic academic settings could help mitigate the long-term behavioral impacts of prolonged screen exposure.

## Appendices

**Table 6:** Questionnaire

Section	Question / Item	Response Format / Options
Background Data	1. Name	
	2. Age	____ years
	3. Gender	Male / Female / Other
	4. Course	
	5. Semester	
	6. Height	____ cm
	7. Weight	____ kg
Details on Screen Time	1a. Total screen time (TV, mobile, laptop, etc.) before lockdown	____ hrs/day
	1b. Has your screen time changed during lockdown?	1) Remained the same 2) Increased 3) Decreased
	1c. If increased or decreased, for how long?	____ hrs/day
	2a. Time spent on social media (Facebook, WhatsApp) before lockdown	____ hrs/day
	2b. Has your social media usage changed during lockdown?	1) Remained the same 2) Increased 3) Decreased
	2c. If increased or decreased, for how long?	____ hrs/day
	3a. OTT platform usage (Amazon, Netflix, etc.) before lockdown	____ hrs/day
	3b. Change during lockdown	1) Remained the same 2) Increased 3) Decreased
	3c. If increased or decreased, for how long?	____ hrs/day
	4a. Gaming duration before lockdown	____ hrs/day
	4b. Change during lockdown	1) Remained the same 2) Increased 3) Decreased
	4c. If increased or decreased, for how long?	____ hrs/day
	4d. Do you feel playing online games reduces stress?	Yes / No
Details on Sleep	Instructions: The following questions relate to your usual sleep habits during the past month only.	
	1. When have you usually gone to bed?	Before lockdown: ____ Lockdown: ____
	2. How long (in minutes) does it take you to fall asleep?	Before lockdown: ____ Lockdown: ____
	3. When have you usually gotten up in the morning?	Before lockdown: ____ Lockdown: ____
	4. How many hours of actual sleep do you get at night?	Before lockdown: ____ Lockdown: ____
	5. During the past month, how often have you had trouble sleeping because of various reasons?	0 = Not during past month 1 = < once/week 2 = 1–2×/week 3 = ≥3×/week
	6. Overall sleep quality before lockdown	Very good (0) Fairly good (1) Fairly bad (2) Very bad (3)
	6A. Overall sleep quality during lockdown	Very good (0) Fairly good (1) Fairly bad (2) Very bad (3)
	7. Use of sleeping medication before lockdown	0 1 2 3 (as above)
	7A. Use of sleeping medication during lockdown	0 1 2 3 (as above)
	8. Trouble staying awake while driving, eating, or socializing before lockdown	0 1 2 3
	8A. During lockdown	0 1 2 3
	9. Difficulty keeping enthusiasm to get things done before lockdown	0 = None 1 = Slight 2 = Somewhat 3 = Very big problem
	9A. During lockdown	0 1 2 3
	10. Bed partner or roommate before lockdown	1) No 2) Other room 3) Same room 4) Same bed
	10A. Bed partner or roommate during lockdown	1) No 2) Other room 3) Same room 4) Same bed
	11. If you have a roommate, how often in the past month have you (as reported by roommate):	0 1 2 3
	a. Loud snoring	
	b. Long pauses between breaths	
	c. Leg twitching or jerking while asleep	
	d. Disorientation or confusion while sleeping	
	e. Other restlessness	
Pittsburgh Sleep Quality Index (PSQI)	1. During the past month, what time have you usually gone to bed at night?	____
	2. How long (in minutes) has it taken you to fall asleep each night?	____
	3. What time have you usually gotten up in the morning?	____
	4. How many hours of actual sleep do you get at night?	____
	5. During the past month, how often have you had trouble sleeping because you: a) Cannot get to sleep within 30 minutes; b) Wake up in the middle of the night or early morning; c) Have to get up to use the bathroom; d) Cannot breathe comfortably; e) Cough or snore loudly; f) Feel too cold; g) Feel too hot; h) Have bad dreams; i) Have pain; j) Other reasons?	0–3 scale (as above)
	6. During the past month, how would you rate your sleep quality overall?	Very good / Fairly good / Fairly bad / Very bad
	7. During the past month, how often have you taken medicine to help you sleep?	0–3
	8. How often have you had trouble staying awake while driving, eating meals, or engaging socially?	0–3
	9. During the past month, how much of a problem has it been for you to keep up enthusiasm?	0–3
	10. Do you have a bed partner or roommate? If yes, how often have they noticed: a) Loud snoring; b) Long pauses between breaths; c) Leg twitching; d) Disorientation; e) Restlessness?	0–3
PHQ-9	Over the last 2 weeks, how often have you been bothered by the following problems?	0 = Not at all 1 = Several days 2 = > half the days 3 = Nearly every day
	1. Little interest or pleasure in doing things	
	2. Feeling down, depressed, or hopeless	
	3. Trouble falling/staying asleep, or sleeping too much	
	4. Feeling tired or having little energy	
	5. Poor appetite or overeating	
	6. Feeling bad about yourself, or that you’re a failure	
	7. Trouble concentrating on things	
	8. Moving or speaking slowly or being fidgety/restless	
	9. Thoughts that you’d be better off dead or of hurting yourself	
PSS-4	In the last month, how often have you:	0 = Never 1 = Almost never 2 = Sometimes 3 = Fairly often 4 = Very often
	1. Felt that you were unable to control important things in your life	
	2. Felt confident about your ability to handle personal problems (R)	
	3. Felt that things were going your way (R)	
	4. Felt difficulties were piling up so high that you could not overcome them	
Fear of COVID-19 Scale (FCV-19S)	Indicate your agreement with each statement	1 = Strongly disagree → 5 = Strongly agree
	1. I am most afraid of coronavirus-19.	
	2. It makes me uncomfortable to think about coronavirus-19.	
	3. My hands become clammy when I think about coronavirus-19.	
	4. I am afraid of losing my life because of coronavirus-19.	
	5. When watching news about coronavirus-19, I become nervous or anxious.	
	6. I cannot sleep because I’m worrying about coronavirus-19.	
	7. My heart races or palpitates when I think about coronavirus-19.	
IGDS9-SF	Please indicate how often the following applies to you:	1 = Never 2 = Rarely 3 = Sometimes 4 = Often 5 = Very often
	1. I feel preoccupied with gaming.	
	2. I feel more irritability, anxiety, or sadness when I cannot play.	
	3. I need to spend increasing amounts of time gaming.	
	4. I unsuccessfully tried to control or stop gaming.	
	5. I lost interest in previous hobbies due to gaming.	
	6. I continue gaming despite knowing problems it causes.	
	7. I deceive family or others about time spent gaming.	
	8. I use gaming to escape negative mood.	
	9. I risk or lose relationships or opportunities because of gaming.	

## References

[1] Singh R, Suri JC, Sharma R, Suri T, Adhikari T (2018). Sleep pattern of adolescents in a school in Delhi, India: Impact on their mood and academic performance. Indian J Pediatr.

[2] Dewald JF, Meijer AM, Oort FJ, Kerkhof GA, Bögels SM (2010). The influence of sleep quality, sleep duration and sleepiness on school performance in children and adolescents: a meta-analytic review. Sleep Med Rev.

[3] Tarokh L, Saletin JM, Carskadon MA (2016). Sleep in adolescence: physiology, cognition and mental health. Neurosci Biobehav Rev.

[4] Jaqua EE, Hanna M, Labib W, Moore C, Matossian V (2023). Common sleep disorders affecting older adults. Perm J.

[5] Hirshkowitz M, Whiton K, Albert SM (2015). National Sleep Foundation's sleep time duration recommendations: methodology and results summary. Sleep Health.

[6] Owens J (2014). Insufficient sleep in adolescents and young adults: an update on causes and consequences. Pediatrics.

[7] Chattu VK, Manzar MD, Kumary S, Burman D, Spence DW, Pandi-Perumal SR (2018). The global problem of insufficient sleep and its serious public health implications. Healthcare (Basel).

[8] Mohan D, Bashingwa JJ, Tiffin N, Dhar D, Mulder N, George A, LeFevre AE (2020). Does having a mobile phone matter? Linking phone access among women to health in India: an exploratory analysis of the National Family Health Survey. PLoS One.

[9] Murugiah P (2020). Internet usage in India: the global analytics (Chapter 4). Measuring and Implementing Altmetrics in Library and Information Science Research.

[10] Claudio MC, Rehany Z, Stachtari K, Guadagno E, Osmanlliu E, Poenaru D (2024). Exploring the digital divide: results of a survey informing mobile application development. Front Digit Health.

[11] Al-Mawali A (2015). Non-communicable diseases: shining a light on cardiovascular disease, Oman’s biggest killer. Oman Med J.

[12] Kelishadi R, Ardalan G, Gheiratmand R (2007). Association of physical activity and dietary behaviours in relation to the body mass index in a national sample of Iranian children and adolescents: CASPIAN study. Bull World Health Organ.

[13] Gray K, Annabell L, Kennedy G (2010). Medical students' use of Facebook to support learning: insights from four case studies. Med Teach.

[14] Farmer AD, Bruckner Holt CE, Cook MJ, Hearing SD (2009). Social networking sites: a novel portal for communication. Postgrad Med J.

[15] Shabur MA, Siddiki MR (2024). Investigating social media's impact on the new era of interactive learning: a case study of Bangladesh. Heliyon.

[16] Abbasi S, Ayoob T, Malik A, Memon SI (2020). Perceptions of students regarding E-learning during Covid-19 at a private medical college. Pak J Med Sci.

[17] Galli F, Reglero G, Bartolini D, Visioli F (2020). Better prepare for the next one. Lifestyle lessons from the COVID-19 pandemic. PharmaNutrition.

[18] Goethals L, Barth N, Guyot J, Hupin D, Celarier T, Bongue B (2020). Impact of home quarantine on physical activity among older adults living at home during the COVID-19 pandemic, qualitative interview study. JMIR Aging.

[19] Altena E, Baglioni C, Espie CA (2020). Dealing with sleep problems during home confinement due to the COVID-19 outbreak: practical recommendations from a task force of the European CBT-I Academy. J Sleep Res.

[20] Chen Q, Liang M, Li Y (2020). Mental health care for medical staff in China during the COVID-19 outbreak. Lancet Psychiatry.

[21] Lalin SA, Ahmed MN, Haq SM (2024). The effects of the COVID-19 pandemic on students' academic performance and mental health: An overview. Reg Sci Pol Prac.

[22] Filip R, Gheorghita Puscaselu R, Anchidin-Norocel L, Dimian M, Savage WK (2022). Global challenges to public health care systems during the COVID-19 pandemic: a review of pandemic measures and problems. J Pers Med.

[23] Hammami A, Harrabi B, Mohr M, Krustrup P (2022). Physical activity and coronavirus disease 2019 (COVID-19): specific recommendations for home-based physical training. Manag Sport Leisure.

[24] Pontes HM, Griffiths MD (2015). Measuring DSM-5 internet gaming disorder: development and validation of a short psychometric scale. Comp Hum Behav.

[25] Buysse DJ, Reynolds 3rd CF, Monk TH, Berman SR, Kupfer DJ (1989). The Pittsburgh Sleep Quality Index: a new instrument for psychiatric practice and research. Psychiatry Res.

[26] Kroenke K, Spitzer RL, Williams JB (2001). The PHQ-9: validity of a brief depression severity measure. J Gen Intern Med.

[27] Ganguly S, Samanta M, Roy P, Chatterjee S, Kaplan DW, Basu B (2013). Patient health questionnaire-9 as an effective tool for screening of depression among Indian adolescents. J Adolesc Health.

[28] Ahorsu DK, Lin CY, Imani V, Saffari M, Griffiths MD, Pakpour AH (2022). The Fear of COVID-19 Scale: development and initial validation. Int J Ment Health Addict.

[29] Lathabhavan R (2023). A psychometric analysis of fear of COVID-19 scale in India. Int J Ment Health Addict.

[30] Cohen S, Williamson G (1988). Perceived stress in a probability sample of the United States. The social psychology of health.

[31] Hedderson MM, Bekelman TA, Li M (2023). Trends in screen time use among children during the COVID-19 pandemic, July 2019 through August 2021. JAMA Netw Open.

[32] Ganesh R, Singh S, Bhargava R, Balhara YP (2022). Screen time and mental well-being of students during the COVID-19 pandemic: findings from a survey among medical and engineering students. Indian J Soc Psych.

[33] Hartley S, Colas des Francs C, Aussert F (2020). The effects of quarantine for SARS-CoV-2 on sleep: an online survey [Article in French]. Encephale.

[34] Helito AC, Lindoso L, Sieczkowska SM (2021). Poor sleep quality and health-related quality of life impact in adolescents with and without chronic immunosuppressive conditions during COVID-19 quarantine. Clinics (Sao Paulo).

[35] Dongol E, Shaker K, Abbas A (2022). Sleep quality, stress level and COVID-19 in university students; the forgotten dimension. Sleep Sci.

[36] McArthur BA, Racine N, McDonald S, Tough S, Madigan S (2023). Child and family factors associated with child mental health and well-being during COVID-19. Eur Child Adolesc Psychiatry.

[37] Cellini N, Canale N, Mioni G, Costa S (2020). Changes in sleep pattern, sense of time and digital media use during COVID-19 lockdown in Italy. J Sleep Res.

